# Laws of Health

**Published:** 1883-03

**Authors:** 


### LAWS OF HEALTH.

MANY persons think it a difficult matter to observe the laws of health; that it requires constant watchfulness and self-denial.

The truth is that whatever we have been accustomed to do regularly, becomes easy; in fact, all habits, good or bad, are hard to change, whether they relate to our thoughts, our diet, dress, methods of business or habits of indolence. The sooner correct methods of thought are formed, the more complete is their blending with the spirit of the individual; there are fewer errors to be cast out and more room for good impressions. There is no truer proverb than that "the boy is father to the man." It is only when they think seriously of these things that parents and teachers can realize their responsibility. Proper training and proper care elevate, the reverse of these degrade.

By proper care, we mean whatever relates to the requirements of the body. Of all the false doctrines that have exploded in the last quarter of a century, that which taught that genius developed best through want and hardship and misery was perhaps the most preposterous.

There may have been rare exceptions, but as a rule we find "a strong mind in a strong body;" and the body can be made strong only through proper and sufficient nourishment, exercise, and dress, and the mind correspondingly developed by correct training.

There is in some children such a thirst for knowledge that great obstacles are overcome to obtain it; and their history proves that they usually struggle up to a higher level intellectually than their less ambitious companions; but who doubts that with better opportunities, better health and fewer obstacles, they would have risen still higher.

